# Supplementary material for: The MYC–NFATC2 axis maintains the cell cycle and mitochondrial function in acute myeloid leukaemia cells
Source: Mol Oncol. 2024 Mar 8;18(9):2234–54. doi: 10.1002/1878-0261.13630 (PMC11467801; doi:10.1002/1878-0261.13630)
Supplement: Supplementary file 1 — Fig. S1. KDM4A transcriptionally regulates NFATC2 in THP‐1 cells. Fig. S2. KDM4A is bound to NFATC2 and NFATC3 in THP‐1 cells. Fig. S3. Apoptosis is not significantly increased in THP‐1 cells after NFATC2 knockdown (KD). Fig. S4. NFATC2 overexpression (OE) in THP‐1 cells leads to downregulation of c‐Myc. [file MOL2-18-2234-s002.zip › Edited_SUPP_FIG_LEGENDS_text.docx]

**Supplementary figures**

**Supplementary Figure 1.** **KDM4A transcriptionally regulates *NFATC2* in THP-1 cells.**

As an expansion of Figure 1, THP-1 cells were transduced with short hairpin RNA (shRNA) constructs non-targeting control (NTC) or *KDM4A*-targeting shRNA (sh*KDM4A* or sh*KDM4A-2*). RNA sequencing (RNA-seq) data were generated from cells harvested 48 hr post-puromycin selection (n=3 biological replicates). RNA-seq data from transduced THP-1 cells were analysed using the Signaling Pathway Impact Analysis (SPIA) pathway enrichment tool. Shown is the expression of genes (from the topmost deregulated ‘Wnt signaling pathway’ from SPIA: see Figure 1A), which had p_adj_<0.05 in the RNA-seq dataset for both NTC vs sh*KDM4A* and NTC vs sh*KDM4A*-2, shown as a heatmap, given as z-scaled Fragments Per Kilobase of transcript per Million mapped reads (FPKM).

**Supplementary Figure 2.** **KDM4A is bound to *NFATC2* and *NFATC3* in THP-1 cells.**

**(A)** As in Figure 1, DNA/protein complexes were immunoprecipitated from untreated THP-1 cells using an anti-KDM4A antibody, and DNA was fragmented and sequenced (n=3). Shown are the KDM4A binding peaks within the *NFATC3* gene region meeting a significance threshold q<0.1, as determined by epic2. **(B)** Data from the TARGET-AML dataset were obtained using *TCGABiolinks* in R. In patient samples from bone marrow (BM) and for which RNA sequencing (RNA-seq) data were available (n=119) were compared for *NFATC2* and *NFATC3* expression. Shown is a scatterplot of expression (in Fragments Per Kilobase of transcript per Million mapped reads (FPKM)) with the Pearson’s correlation coefficient and *p* value of *NFATC2*–*NFATC3* correlation.

**Supplementary Figure 3. Apoptosis is not significantly increased in THP-1 cells after *NFATC2* knockdown (KD).**

THP-1 cells transduced with either non-targeting control (NTC) or short hairpin RNA targeting *NFATC2* (sh*NFATC2*-1 or -2) were stained with annexin-FITC/APC and DAPI, at either 72 hr **(A)** or 144 hr **(B)** post-puromycin selection. Shown are the mean % values of Annexin^+^ cells in each shRNA transduction group (n=3), with the associated one-way analysis of variance (ANOVA) result for an across-group comparison (ns = not significant; p>0.05).

**Supplementary Figure 4. *NFATC2* overexpression (OE) in THP-1 cells leads to downregulation of c-Myc.**

**(A)** *NFATC2* expression measured in THP-1 cells expressing either of the vectors Empty or h*MYC* using quantitative real time polymerase chain reaction (qRT-PCR), shown as mean -log_2_ fold changes compared to Empty (n=3). A two-sided, unpaired t-test for a difference in means was used (*p=0.039). **(B)** *NFATC2* expression measured in short hairpin RNA (shRNA)-transduced THP-1 cells using qRT-PCR at 48 hr post-puromycin selection. Differential expression as compared between either non-targeting control (NTC) vs. *MYC*-targeting shRNA 1 (sh*MYC*-1) and NTC vs. sh*MYC*-2 shown as mean -log_2_ fold changes (n=3). A one-way analysis of variance (ANOVA) for an across-group difference in means was used and the overall p value is shown (ns = not significant; p>0.05). **(C)** c-Myc protein expression was measured by immunoblot in THP-1 cells expressing either of the vectors Empty, h*NFATC2*. Quantitative densitometry results are shown (n=3), expression relative to total histone 3 (H3), and the representative immunoblot is shown. A two-sided, unpaired t-test for a difference in means was used (**p=0.006).
