# Supplementary figures and images for: The MYC–NFATC2 axis maintains the cell cycle and mitochondrial function in acute myeloid leukaemia cells
Source: Mol Oncol. 2024 Mar 8;18(9):2234–54. doi: 10.1002/1878-0261.13630 (PMC11467801; doi:10.1002/1878-0261.13630)

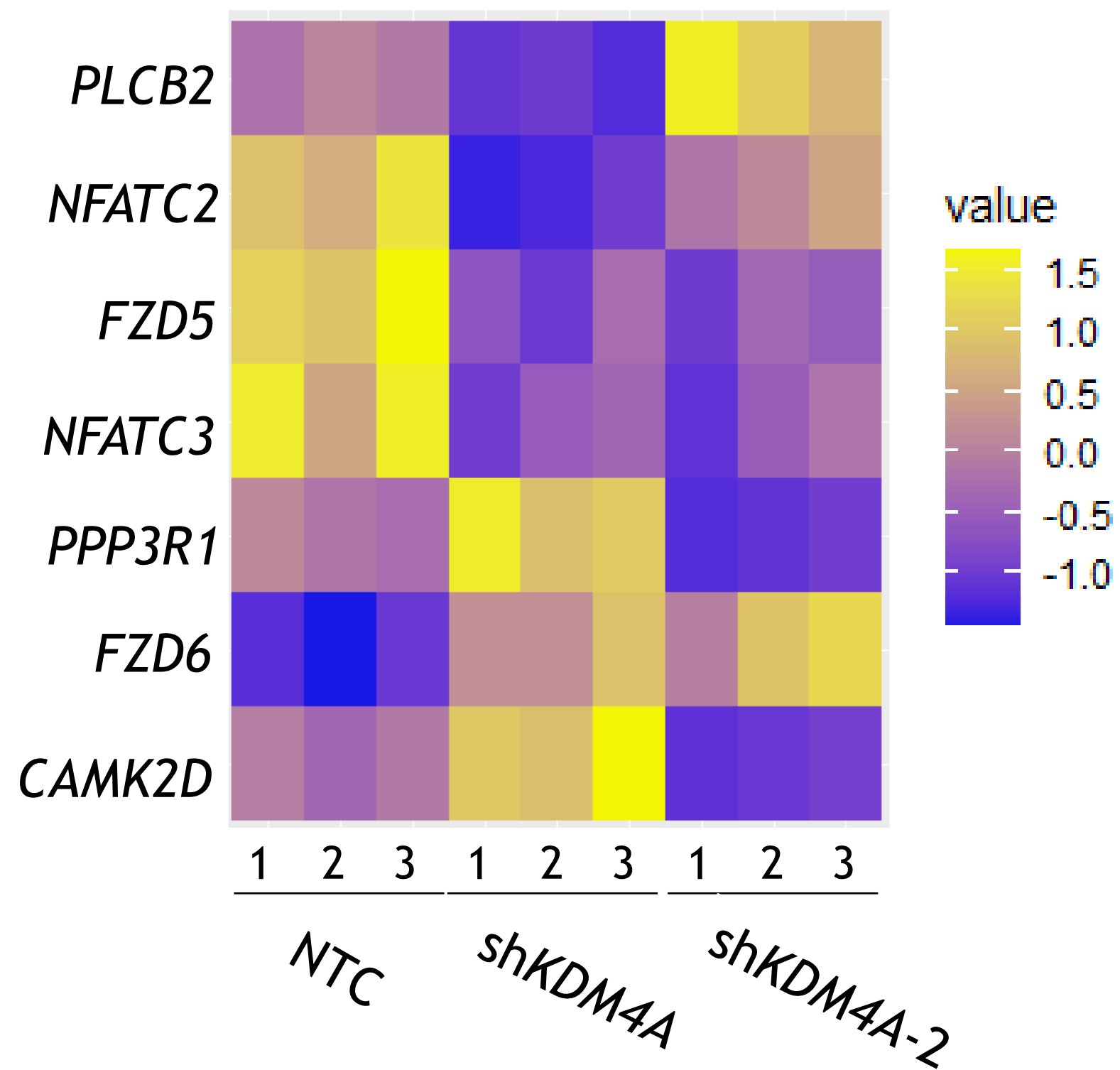

Supplement: Supplementary file 1 — Fig. S1. KDM4A transcriptionally regulates NFATC2 in THP‐1 cells. Fig. S2. KDM4A is bound to NFATC2 and NFATC3 in THP‐1 cells. Fig. S3. Apoptosis is not significantly increased in THP‐1 cells after NFATC2 knockdown (KD). Fig. S4. NFATC2 overexpression (OE) in THP‐1 cells leads to downregulation of c‐Myc. [file MOL2-18-2234-s002.zip › SUPP_FIGURE_1.pdf]

A

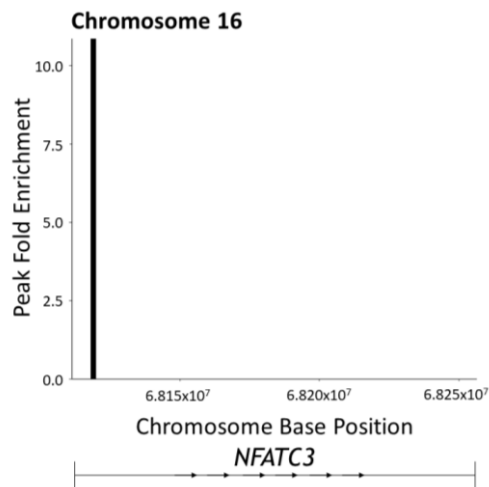

B

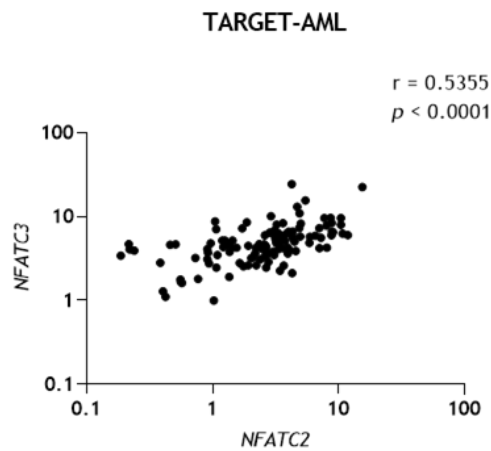

Supplement: Supplementary file 1 — Fig. S1. KDM4A transcriptionally regulates NFATC2 in THP‐1 cells. Fig. S2. KDM4A is bound to NFATC2 and NFATC3 in THP‐1 cells. Fig. S3. Apoptosis is not significantly increased in THP‐1 cells after NFATC2 knockdown (KD). Fig. S4. NFATC2 overexpression (OE) in THP‐1 cells leads to downregulation of c‐Myc. [file MOL2-18-2234-s002.zip › SUPP_FIGURE_2.pdf]

A

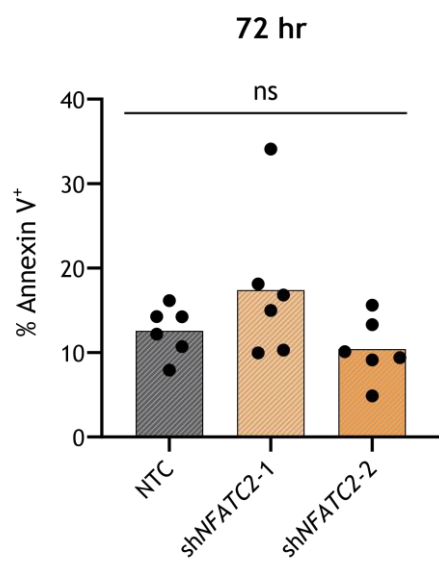

B

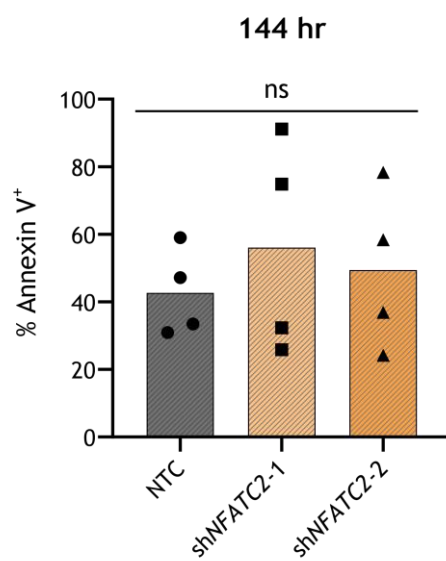

Supplement: Supplementary file 1 — Fig. S1. KDM4A transcriptionally regulates NFATC2 in THP‐1 cells. Fig. S2. KDM4A is bound to NFATC2 and NFATC3 in THP‐1 cells. Fig. S3. Apoptosis is not significantly increased in THP‐1 cells after NFATC2 knockdown (KD). Fig. S4. NFATC2 overexpression (OE) in THP‐1 cells leads to downregulation of c‐Myc. [file MOL2-18-2234-s002.zip › SUPP_FIGURE_3.pdf]

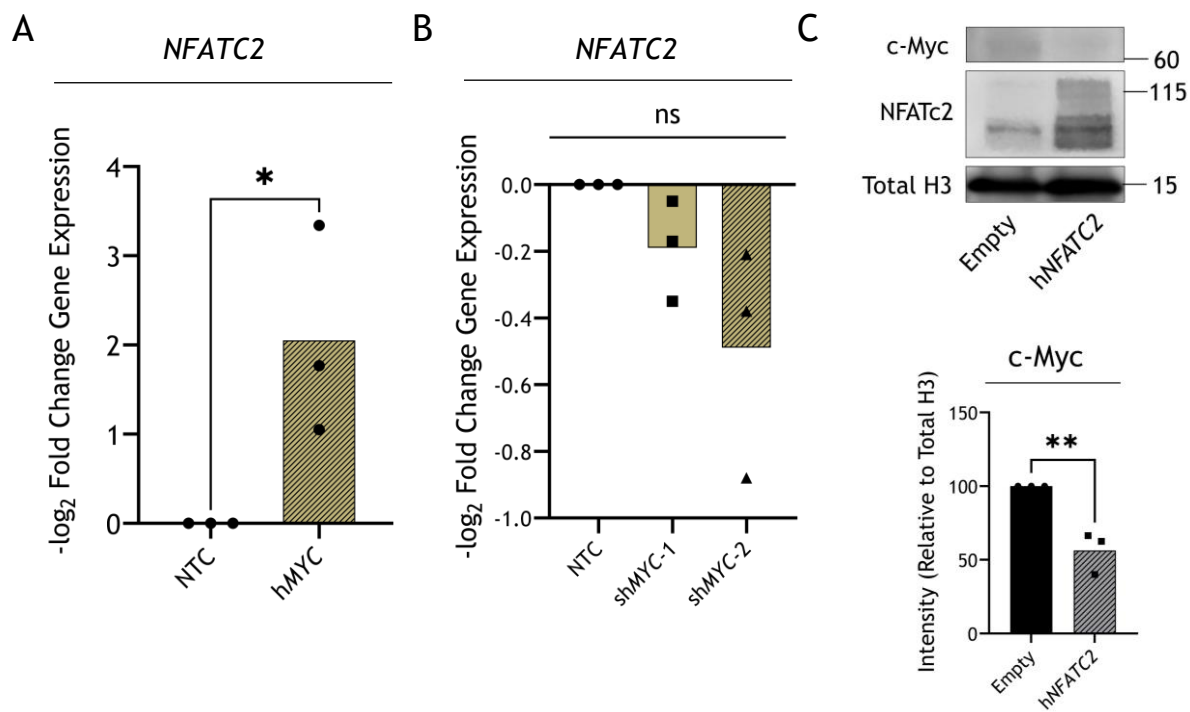

Supplement: Supplementary file 1 — Fig. S1. KDM4A transcriptionally regulates NFATC2 in THP‐1 cells. Fig. S2. KDM4A is bound to NFATC2 and NFATC3 in THP‐1 cells. Fig. S3. Apoptosis is not significantly increased in THP‐1 cells after NFATC2 knockdown (KD). Fig. S4. NFATC2 overexpression (OE) in THP‐1 cells leads to downregulation of c‐Myc. [file MOL2-18-2234-s002.zip › SUPP_FIGURE_4.pdf]
