# Supplementary material for: The MYC–NFATC2 axis maintains the cell cycle and mitochondrial function in acute myeloid leukaemia cells
Source: Mol Oncol. 2024 Mar 8;18(9):2234–54. doi: 10.1002/1878-0261.13630 (PMC11467801; doi:10.1002/1878-0261.13630)
Supplement: Supplementary file 2 — Table S1. Primer sequences used in the study. Table S2. Western blotting antibodies used in the study. Table S3. Five Gene Set Enrichment Analysis (GSEA) pathways were enriched in the data for NFATC2‐targeting short hairpin RNA (shNFATC2) knockdown (KD) in THP‐1 cells. [file MOL2-18-2234-s001.docx]

**Supplementary Tables**

| **Primer Target** | **Forward Primer Sequence** | **Reverse Primer Sequence** |
| --- | --- | --- |
| *NFATC2* | ACCCTTGGAGCCCAAAAACA | CTTTCCGCAGCTCAATGTCG |
| *GAPDH* | GTCAACGGATTTGGTCGTATTG | TGTAGTTGAGGTCAATGAAGGG |
| *ACTB* | CACAGAGCCTCGCCTTT | GCGGCGATATCATCATC |
| *MYC* | CAAGAGGCGAACACACAACG | CAACTCCGGGATCTGGTCAC |
| *CCNA2* | TGGCGGTACTGAAGTCCGG | CAAGGAGGAACGGTGACATGC |
| *CCNB1* | CAGCTCTTGGGGACATTGGTAAC | ACTGGCACCAGCATAGGTACC |
| *CCND1* | GATCAAGTGTGACCCGGACTG | CCTTGGGGTCCATGTTCTGC |
| *CCND2* | ACCAACACAGACGTGGATTGT | CTCCGACTTGGATCCGTCAC |
| *CCND3* | CCTCCTACTTCCAGTGCGTG | AGGCCAGGAAATCATGTGCA |
| *CCNE1* | CAACGTGCAAGCCTCGGA | AAAGTGCTGATCCCTTAAGTATGTC |
| *CCNE2* | ATCCTTCACCTTTGCCTGATTT | CCTCATCTGTGGTTCCAAGTCA |

**Supplementary Table 1. Primer sequences used in the study.**

Shown are the forward and reverse primer sequences used for quantitative real time polymerase chain reaction (qRT-PCR) in this study, per target gene.

| **Target** | **Species** | **Clone** | **Supplier** | **Catalogue ID** |
| --- | --- | --- | --- | --- |
| NFATc2 | Rabbit | D43B1 | CST | 5861 |
| Histone 3 | Mouse | 1B1B2 | CST | 14269 |
| Β-actin | Mouse | 8H10D10 | CST | 3700 |
| COX IV | Rabbit | 3E11 | CST | 4850 |
| c-Myc | Rabbit | Y69 | Abcam | ab32072 |

**Supplementary Table 2. Western blotting antibodies used in the study.**

Each of the Western blotting antibodies used in this study is shown with the information regarding species isotype, clone, supplier and catalogue ID.

| **Pathway** | **sh*NFATC2*-143** | | **sh*NFATC2*-146** | |
| --- | --- | --- | --- | --- |
|  | **NES** | **FDR q value** | **NES** | **FDR q value** |
| [HALLMARK_MYC_TARGETS_V1](http://www.gsea-msigdb.org/gsea/msigdb/cards/HALLMARK_MYC_TARGETS_V1) | 2.69 | <0.001 | 2.68 | <0.001 |
| [HALLMARK_MYC_TARGETS_V2](http://www.gsea-msigdb.org/gsea/msigdb/cards/HALLMARK_MYC_TARGETS_V2) | 2.17 | <0.001 | 2.13 | 0.001 |
| [HALLMARK_OXIDATIVE_PHOSPHORYLATION](http://www.gsea-msigdb.org/gsea/msigdb/cards/HALLMARK_OXIDATIVE_PHOSPHORYLATION) | 2.11 | <0.001 | 2.49 | <0.001 |
| [HALLMARK_DNA_REPAIR](http://www.gsea-msigdb.org/gsea/msigdb/cards/HALLMARK_DNA_REPAIR) | 1.67 | 0.010 | 1.94 | <0.001 |

**Supplementary Table 3. Five Gene Set Enrichment Analysis (GSEA) pathways were enriched in the data for *NFATC2*-targeting short hairpin RNA (sh*NFATC2*) knockdown (KD) in THP-1 cells.** Following RNA-sequencing (RNA-seq) in THP-1 cells transduced with short hairpin RNA (shRNA) targeting *NFATC2* (sh*NFATC2*-1 or sh*NFATC2*-2), or non-targeting control (NTC), GSEA was used to identify enriched pathways within the perturbed genes. Shown are the GSEA pathways for which the q-value was <0.05 for THP-1 both sh*NFATC2*-1 vs. NTC and sh*NFATC2*-2 vs. NTC, alongside the associated statistics: NES = normalised enrichment statistics; FDR = false discovery rate.
